# Medical and Physical Intelligence

**Published:** 1802-09-01

**Authors:** 


### MEDICAL AND PHYSICAL INTELLIGENCE.

### [ FOREIGN AND DOMESTIC. ]

*Preliminary Observations contained in the Program of the Prize proposed by the Society of Medicine of Bourdeaux, in its public Sitting of the 27 Fructidor, Year 9.*

Hippocrates is, without contradiction, the greateſt medical genius of antiquity; his writings, for a length of ages, have been always honoured by true phyſicians, always imprinted with the veneration of the wiſe, always preſerved with a religious attention. By an unanimous aſſent, by the univerſal voice, Hippocrates is proclaimed, in all times and in all places, the oracle, the father, and the god of Medicine. Of all the ſtatues erected by the antients to great men, his is the moſt inviolate, and the moſt revered. His works breathe candour and frankneſs; they are eternal teſtimonies of the depth of his genius, of his veracity, of his modeſty, and of the cares which he every where takes to form a virtuous phyſician. Laſtly, we only perceive in his ſoul one ſentiment, the love of good; and in the courſe of his long life, one ſingle deed, the relief of the ſick. Happy the people, if the ſucceſſors of that ſupreme legiſlator had always preſerved his doctrine, as they kept at Rome, the fire of Veſta!

But too often phyſicians, ſmitten with the love of ſyſtems, have oſt themſelves in the bottomleſs abyſs of conjecture; they have wiſhed too often to ſubſtitute the product of their imagination to the true laws of Nature. How many ſects do we find in Medicine? (Should there any exiſt in the art of healing?) How many theories alternately adopted and rejected? Galen abandons himſelf to diſcuſſions more ingenious than uſeful; the Arabs refine on the ſubtleties of Galen; the alchymiſts burn the works of the ancients; then the follies of aſtrology, of geomancy, of cabaliſtical learning, were introduced into medicine. In all the crucibles are ſought the *fountain of youth, the elixir of long life, and the stone of immortality.* In the midſt of this univerſal delirium, Hippocrates remains alone, upright, as a column not to be ſhaken; the works of this great man are read, ſtudied, commented upon. Valleſius, Baillou, Houlier, Duret, make numerous diſciples; the Hippocratic medicine ſhines again in all its luſtre, and the Temple of Cos becomes a ſacred ark, out of which there is no ſafety.

Many learned men have made the catalogue of the writings of Hippocrates; all cannot have an equal right to our confidence. Galen among the ancients, and Gruner among the moderns, have ſhewn much criticiſm and exactitude on this ſubject; and from theſe two phyſicians, the legitimate works of the father of medicine cine are known to us. It is in thefe treatifes, that the genius of Hippocrates is included, all intire; it is in thofe immortal writings, commented on by fo many great practitioners, fhining with a luftre that twenty-two centuries have not tarnifhed, that we find the doctrine of the firft and moft fkilful legiflator of the art; there we fee the extent of his knowledge in the different parts of Clinical medicine. In effect, what depth in his aphorifms! What fagacity in his book *of airs, of waters, and of places.* What accurate notions on the temperature of the different feafons of the year; on the pofition of cities; on the maladies to which they are expofed; on the manner of living of the men who have fixed their abode there. What judgment in his *Treatise of Prenotions!* What candour and ability in his *History of Epidemicals!* What wifdom in his book of *Diet in acute Diseases!* It is apparently the God of Medicine pronouncing oracles; it is Nature herfelf dictating her laws, by the mouth of the moft worthy Interpreter fhe ever had.

It is very aftonifhing, that among the commentators of this great man, not one has been found who has prefented us with his doctrine in all its *ensemble,* with that feries, that concatenation of ideas which makes them one illuftrate another; and laftly, with that order which includes in particular divifions (but with that coup d'oeil of genius, which, as Montefquieu fays of Tacitus, abridges every thing becaufe it fees every thing) what the Sage of Cos has told us on the figns, on the prenotions, on the caufes, on the curation, on the regimen, on the maladies of different ages, on the aphoriftic art, on the epidemicals, on medical meteorology, on different points of chirurgy, &c. &c. Would not an admirable whole refult from this affemblage? And of thefe different traits, formed by a fkilful artift, fhould we not have at length what we might call the *tableau of Hippocratic medicine?* It is precifely this *tableau,* the execution of which the Society now calls for.

But however well this work might be done, it fhould be included within reafonable limits. The Society of Medicine does not require the eulogium of Hippocrates; this eulogium is already in the mouth of all true phyficians; neither does it defire a notice on his life; what we want the moft in that of illuftrious men, is the hiftory of their writings. Neither does the Society demand a new catalogue of his works, nor a differtation on thofe which are his, and thofe which do not belong to him. Gruner accomplifhed this laft tafk in 1772; and certainly, it would be difficult to find a criticifm more judicious, and a philology more vaft, than of that learned phyfician. Neither, laftly, does it want a *commentary* of his works; who could comment on them better than Vallefius, than Profper Martian, than Gorter, than Coray, and fo many others?

From an union of all thefe motives, the Society of Medicine of Bourdeaux is induced to offer a prize of the value of 300 francs, to the perfon who fhall prefent with order, regularity, and method, the *Ensemble of the Doctrine of Hippocrates,* or the *Tableau of the Hippocratic Medicine.* The prize will be adjudged in the public fitting of Fructidor, year 10. The Memoirs to be received till the

1ft Ther-

1ſt Thermidor, of the ſame year. They ſhould be addreſſed carriage free, to the Secretary General of the Society. The authors are incited to join a ſealed billet, containing a motto, with their name and addreſs. The billets only will be opened, which will be joined either to the Memoir that ſhall have gained the prize, or to that which ſhall approach the neareſt to it. The reſiding members of the Society of Medicine are excluded from the *concours*.

---

*Description of a new Species of Phaca, by* Citizen CLARION. *Phaca glabra. P. caule ramoso prostrato, foliis ovato-lanceolatis, florum alis integerrimis, leguminibus glabris.*

The root of this plant is vivacious, apparently ligneous, ſimple or bifurcated, a little fibrous; the neck (collet) gives riſe to many ſtalks, rude, expanded, chamfered, ſingle below, and branching towards the top; the leaves are alternate, few in number, pennated in odd numbers *(avec impaire)*; the common *petiole* bears from nine to thirteen ſmall oval leaves, terminated by a point rather ſaliant and as it were glandulous; of a yellowiſh green under; the ſtipules are oppoſite, oval, acute, ſometimes united, and then they ſheath the ſtalk; the peduncles over-paſs the leaves and carry an ear of horizontal flowers or *penchees;* the calyx has five teeth, and is covered with black down or hairs; the corolla is papillonaceous white; with the exception of the *carene*, and of the part of the wings near to the *carene*, which are violet coloured. The etendard is oval, chamfered, elevated behind; the wings are oval-linear, curved, ſhorter than the ſtandard *(etendard)*. The ovary is ſupported on a ſhort pedicle, and is ſurmounted with a ſtyle, curved ſemicircular, and terminated by a flat ſtigma. To this piſtil ſucceeds a cod or huſk glabrous, pediculated, veſiculous, pointed at the two extremities; the upper ſuture enters again a little within the huſk and bears reniform grains or ſeeds. The tumour glabrous Phaca differs from the Phaca Gerardi viii. by its glabrous huſk; from the Alpine Phaca by its upright ſtalk; and from the Phaca Auſtralis by its entire wings. It grows in the mountains of Pray, department of lower Alps. It flowers in Meſſidor.

---

*On a new Genus of Inſects, named Atracto-Ceros.*

This inſect has been brought from the kingdom of Oware, in Africa, by Cit. Paliſſot Beauvois. The name which this voyager gives it, ſignifies *antenne-fuseau.*

Agreeably to its form, it is diſtinguiſhed from other coleopteres, in having wings much longer than its etwees, and which do not fold back under them, and in having five articulations to all the tarſi. This laſt character approaches to the *staphilines*, while the preceding one bears analogy to the *necidales*. The form of its antennulæ is very ſingular. Cit. Beauvois thinks that this inſect lives in the woods.

---

*Extract*

*Extract of a Memoir on two Species of non descript oviparous Quadrupeds, lately read to the National Institute of France, by Citizen* LACEPEDE.

We find among reptiles, all the combinations of digits, from five to one, taken between two pairs of hands or claws. There wanted to these combinations those of four digits, or two digits, or one single digit to each of its four claws. The two species which Cit. Lacepede describes, fill up two of these three desiderata. One has four digits to each foot; he names it *tetradactyle ;* the other has only one, he calls it *monodactyle.* These two lizards ought to establish two new sub-genera in the genus of lizards; or, according to the method of Cit. Lacepede, they ought to pertain to the chalcide genus of the natural method proposed by Cit. Alex. Bronguiart.

The chalcide tetradactyle has the four hands very small, and so short, that they can hardly reach the earth; and indeed, it does not use its hands to advance, it creeps in the manner of serpents; the first and the fourth digits are so small, that they are perceived with difficulty; the third, on the contrary, is ve y long. The body is slender, cylindrical; the tail is three or four times longer than the body; the scutcheons of the head are pretty nearly disposed, like those of adders. The tongue is flat, broad, but short, and somewhat round towards the end. A sort of furrow is excavated on each side of the animal, from the angle of the chops to the hind foot. The upper scales of the neck and body are almost square, raised by and disposed in rings; it was two decimetres and nine centimetres in length.

The chalcide monodactyle has the legs still shorter and weaker than those of the tetradactyle; only one single digit is found to each foot. This chalcide is also very long, very slender, cylindrical, and rather resembles an animal of the family of Ophidians than a Saurien reptile. The tongue is round at the end. The upper and under part of the body and of the tail are furnished with scales, long, pointed, and raised. These scales, which anticipate laterally on one another, form transversal rows, partly placed one above another, and apparently festooned. This reptile was four decimetres eight centimetres in its total length.

Cit. Lacepde terminates this memoir, by remarking, that the hollow tubercles which we find disposed in one row under the thighs of lizards of the gecko genus, do not constantly appear in all the individuals of the same species. He cannot yet assign the reason of this singular difference. He concludes from hence, that the distinctive characters of the species which he has named gecko and geckotte, as they cannot be taken from the presence or the absence of those turbercles of the thighs, ought to be drawn from the form of the tubercles of the head and of the body, which are equally globulous in the geckos, while they always represent a small pyramid with three faces in the geckottes.

---

Citizen

Citizen Percy, an Associate Member of the National Institute, lately read in one of its sittings, some Medical and Philosophical Observations on an universal Ankilosis, or motionless state of body in all the joints, and shewed the Class a skeleton of the unfortunate person who lived twelve years in that state, worse than death; of which, according to the expression of Cit. Percy, it is a terrible image, and a long forerunner. This surgeon in chief of the armies, after having cited the ankiloses observed by different physicians, and especially by Real-Colomb, and Cit. Portal, informed the Class that Cit. Francois Maurice Marcien de Simorre, an old officer, had contracted in the campaigns of Corsica, a gouty rheumatism, which took from him successively the use of his fingers, his hands, and his feet, and which, after excessive pains, deprived him of all motion, even of that of the lower jaw, and caused him to lose his sight. He spent many years in an armed chair, without obtaining a moment's sleep, notwithstanding the strongest doses of opium. Reduced to a state wherein he could only suck a little broth or wine, through the very small interval left between the upper and lower teeth, he had two incisive teeth pulled out, and by favour of the opening which he procured by this operation he could speak more freely, and could imbibe liquids with a pipe, and even swallow a little hashed meat. His body, a sort of animated statue or living carcase, formed only one piece; all his bones were soldered into one another; and notwithstanding this extreme pain, the conversation of Simorre was often very gay, and he dictated every year an almanack in verse, which was eagerly bought up to relieve his misery, without hurting his delicacy. That of the year 5 is remarkable for this motto:

> Privé de la lumière, perclus de tout son corps,

> Il se rit de la vie, en attendant la mort.

Simorre had however a physiognomy full of expression, and even of hilarity. The muscles of his face had acquired a singular mobility, they were incessantly in action, either to supply the gestures which he could no longer make, or to shrivel up the skin and drive away the insects that came to sting it. Cit. Percy has developed the original and the progress of this fortunately very rare disease; has examined into its causes, explained the alterations of the bones, and in particular, those of the joints of Simorre, whose skeleton, a monument at once frightful and valuable, of the human figure, is now deposited in the Conservatory at the School of Medicine of Paris.

In a second Memoir, Cit. Percy has described all the effects of a monstrous voracity, to which he has given the name of *Polyphagy.* A young man of the neighbourhood of Lyons, and who had early followed a troop of *batelcurs*, had habituated himself to swallow pebbles, enormous masses of rejected meats, panniers of unripe fruit, and even living animals. Some dreadful accidents and terrible cholics could not make him quit a dangerous habit, which soon became an imperious necessity. Enrolled in the beginning of the last war in one of the battalions of the army of the Rhine, he fought fought near an ambulatory hofpital the aliments which were necef-fary to him. The reliques of the kitchen, the rejected fragments of the diftributions, corrupted meats, were not fufficient. often went to fnatch from the vileft animals the moft difgufting meat; he was conftantly hunting after cats, dogs, and ferpents, which he devoured alive. They were obliged to drive him away, by threats or force, from the chamber of the dead, and from places where the blood drawn from fick perfons was depofited. Attempts were repeatedly made to cure him, by giving him alternately, fat fubftances, acids, opium, and even de la coque du Levant. The difappearance of a child fixteen months old, having excited frightful fufpicions againft him, he took to flight. But in the year 6, he entered into the Infirmary of Verfailles in a con-fumptive ftate, which had fucceeded to his horrible appetite, and which, according to him, proceeded from a filver fork which had ftuck in the inteftinal canal. He died in a fhort time after.

Cit. Teffier, furgeon in chief of that Infirmary, having had the courage to open his body, notwithftanding the infupportable ftench which it emitted, could not find the fork. The ftomach was of an extraordinary amplitude; the inteftines, quite ulcerated, exhibited remarkable renflemens, and the veficle of the gall was of large dimenfions. Tarare was fhort in fize, feeble, and infirm; his afpect had nothing ferocious. When he was fafting, the fkin of his belly could almoft encircle his body; and when he was fed he appeared as if dropfical; a thick vapour iffued in torrents from his mouth; all his whole body fmoaked; fweat ran copioufly from his head; and, like moft voracious animals, he s'assou piffoirt to digeft.

Cit. Pėrcy has terminated his memoir by the expofition of the interior organization of the unhappy perfons condemned by nature to this exorbitant and cruel appetite; he has explained moft of the phenomena which they prefent; and concludes from the numerous examples of Polyphagy which he has collected, that the unhappy fubjects of it moft frequently find the end of their miferies in death before the age of forty years.

---

William Blair, A. M. Member of the Royal College of Sur-geons, Fellow of the Medical Societies in London and Paris, Sur-geon of the Locke Hofpital and Afylum, and of the Bloomsbury Difpenfary, Lecturer on the Animal Economy, and on the Clinical Practice of Surgery, will fhortly publifh an Entire Syftem of Medical and Operative Surgery; comprifing the lateft improvements in Theory and Practice; with numerous Engravings of Chirurgical Difeafes, Operations, and Inftruments. Dedicated to the Royal College of Surgeons in London.

---

Dr. Wittman, of the Royal Artillery, who went from England to Turkey as Surgeon and Medical Affiftant to the Military Miffion under the command of General Koehler, is preparing for publication an Account of his interefting Travels; to which he will annex Copious Obfervations on the Plague and on the Dif-eafes eases of the Turks. His residence of nearly three years in the Turkish army, and his appointment as Physician to the Grand Vizier, qualify him in a peculiar manner for the task he has undertaken.

